# Microbial community succession along the entrance to distant zone in karst cave ecosystem: community assembly and distance decay patterns in weathered rock and sediment habitats

**DOI:** 10.1186/s40793-026-00900-z

**Published:** 2026-04-24

**Authors:** Jiaming Du, Liyuan Ma, Xinping Huang, Xiaolu Lu, Hongmei Wang

**Affiliations:** 1 Hubei Key Laboratory of Wetland Evolution and Ecological Restoration, School of Environmental Studies, China University of Geosciences, Wuhan, 430074 China; 2 Science Faculty, University of Waterloo, Waterloo, N2L 3G1 Canada; 3 State Key Laboratory of Geomicrobiology and Environmental Changes, China University of Geosciences, Wuhan, 430074 China

**Keywords:** Karst caves, Weathered rock, Sediment, Cave microbes, Community assembly, Distance decay

## Abstract

**Background:**

Microbial communities in karst caves represent highly specialized and distinct assemblages shaped by the unique physicochemical properties of cave environments. Although recent research has advanced our understanding of community composition, the underlying assembly mechanisms and spatial structuring processes, including distance decay relationships, remain insufficiently resolved. The aim of this research was to elucidate the community assembly mechanisms and distance decay patterns of microbial communities in weathered rock and sediment habitats.

**Results:**

We analyzed 16S rRNA gene sequencing data from 333 weathered rock and sediment samples collected across karst caves in the southwestern region of China, India, Italy, and Mexico. Distinct microbial communities were observed between weathered rock and sediment habitats, with sediment communities showing higher alpha diversity and more pronounced compositional shifts with increasing distance from the entrance. Both weathered rock and sediment communities showed distance-decay relationships, suggesting that geographic distance contributes to microbial community differentiation in caves. Moreover, stochastic processes, particularly dispersal limitation, play a dominant role in the assembly of microbial communities in both weathered rock and sediment. However, the influence of ecological drift is more prominent at local scales but declines at larger spatial scales. Sediment habitat and photic zone have higher connectivity, meaning the stable subgroups were formed to enhance ecological adaptability.

**Conclusion:**

This study revealed that microbial communities in karst caves are strongly shaped by habitat type, the distance from the cave entrance, and spatial scale. The observed distance-decay patterns indicate that geographic distance contributes to microbial community differentiation in caves, but its influence is weaker than that of local environmental and habitat-specific controls. Stochastic processes dominate community assembly, with dispersal limitation as the key mechanism. The findings provide new insights into the ecological mechanisms underlying microbial distribution in extreme subsurface environments and highlight the importance of integrating large-scale datasets for understanding microbial biogeography in karst systems.

## Introduction

Karst caves are unique subterranean landform distributed globally. Weathered rock and surface sediment represent two distinct habitats with contrasting physicochemical properties in karst caves. Weathered rock surfaces typically develop directly on the carbonate bedrock [1], presenting a hard, oligotrophic interface characterized by high calcium concentrations and alkalinity [2]. These surfaces often impose strong environmental filtering due to limited nutrient availability and low permeability. In contrast, cave surface sediments function as nutrient sinks [3], harbor diverse microbial communities that are instrumental in nutrient cycling and the degradation of pollutants [4]. Microbial community species abundance differs between the weathered rock and sediment habitats, with a lower bacterial biomass of about 10^6^ cells g^−1^ in the weathered rock habitats [5], and the phyla *Pseudomonadota* (previously *Proteobacteria*), *Acidobacteriota* (previously *Acidobacteria*), and *Actinomycetota* (previously *Actinobacteria*) were reported to dominate in several weathered rock samples [6–9]. Microbial communities in sediment samples were more complex than in weathered rock and were closer to those in cave soils [10, 11].

The unique composition of microbial communities in different caves is closely related to environmental factors [12, 13]. pH has been proven to play a dominant role in the formation of microbial communities in Heshang Cave [6]. And factors such as total organic carbon (TOC) and metal ions, in terms of both their composition and concentration, have also been found to have significant effects on microbial communities in some karst cave habitats [14, 15]. Moreover, previous studies revealed that there are obvious distinctions in the microbial community structure of the secondary sediments between the entrance region of Baeg-nyong Cave and more distant parts of the cave [16]. Besides, light condition changes the diversity and function of cave microbial communities in caves [17, 18]. Environmentally driven patterns are typically interpreted as deterministic processes within metacommunity theory and represent predictable, niche-based mechanisms such as environmental filtering and biotic interactions [19, 20], in contrast to stochastic processes that emphasize random dispersal, ecological drift, and demographic stochasticity, as described by the neutral paradigm [21, 22]. Increasing evidence suggests that both processes jointly regulate microbial community assembly, with their relative importance varying across ecosystems and spatial scales [20, 23, 24]. Despite growing insights from soil microbial studies, the community assembly mechanisms governing microbial communities in karst caves remain poorly understood, particularly given the distinct environmental constraints of cave systems compared to surface soils [25–27].

To comprehensively understand community assembly dynamics, metacommunity theory provides a robust conceptual framework that links local microbial community composition to regional spatial scales [28]. In this context, a metacommunity is defined as a set of local communities that are linked by dispersal of potentially interacting species [29]. For karst ecosystems, caves can be viewed as insular, island-like habitats embedded within a geological matrix [30]. Metacommunity theory posits that community structure is determined by the interplay between local deterministic factors and regional stochastic factors [31]. Therefore, analyzing distance-decay relationships alongside community assembly mechanisms allows for a holistic assessment of how microbial diversity is maintained across spatially fragmented subsurface habitats.

Investigating microbial diversity and distribution patterns at larger spatial scales can enhance our understanding of the importance of microbial communities and specific taxa in predicting regional and global ecological processes [32]. For example, global surveys of soil eukaryotes have clarified the relative importance of ecological processes and environmental drivers in shaping broad-scale biogeographic patterns by leveraging metagenomics and metabarcoding of global topsoil samples [33]. Moreover, accumulating evidence suggests that the relative contributions of microorganisms to nutrient cycling vary across spatial scales, highlighting the need to link community patterns with ecosystem functioning across regions [34]. In karst cave ecosystems, microbial studies have largely emphasized community composition and environmentally associated variation, either within individual caves or among multiple caves within a given geographic area [35, 36]. Similarly, investigations of caves in the Alpine Mountains have reported diverse archaeal taxa in secondary cave sediments [37]. Nevertheless, most existing cave microbiome studies remain geographically constrained and focus on local- to regional-scale patterns, whereas broader, cross-region syntheses are still limited. Therefore, there is increasing interest in characterizing microbial communities in cave weathered rock and sediment across broader spatial scales to evaluate the generality of observed patterns and the extent to which environmental filtering and spatial processes jointly structure these communities.

While the Earth Microbiome Project (EMP) database contains abundant environmental samples such as soil and seawater, the availability of karst cave samples is limited. Current global studies on caves primarily rely on evaluating sequenced data results, often presented in the form of review articles due to the lack of standardized amplification processing methods [38–40]. Consequently, further investigations into microbial interaction relationships and community assembly mechanisms remain challenging. However, it has been demonstrated that a more comprehensive analysis of microbial communities can be achieved by applying consistent upstream analysis and processing to raw amplification data from published literature [41].

To bridge the gap between local case studies and large-scale microbial biogeography in subsurface environments, we collected weathered rock and sediment samples from karst caves in Guangxi Province, including Luohandu, Panlongdong, and Xincuntun Caves, and microbiome data from weathered rock and sediment samples of karst caves in China, India, Italy, and Mexico were incorporated to broaden the microbial dataset. Using bioinformatics tools and comparing our data with publicly available datasets, the distance decay relationships of microbial communities in weathered rock and sediment habitats were analyzed, while accounting for variations in the distance from the cave entrance. The primary objective of this study is to (1) elucidate the community assembly mechanisms and (2) distance decay relationships of microbial communities in caves on a global scale, thereby advancing our understanding of microbial community formation and their responses to the distance from the cave entrance within karst cave ecosystems.

## Materials and methods

### Data collection of karst cave microbiomes

In total, microbial community data from 15 karst caves were included in this study, obtained from three sources: (i) samples collected from 3 caves in Guangxi Province, China; (ii) samples from 8 caves provided by collaborating institutions; and (iii) publicly available datasets from 4 caves in other regions.

**Fig. 1:**
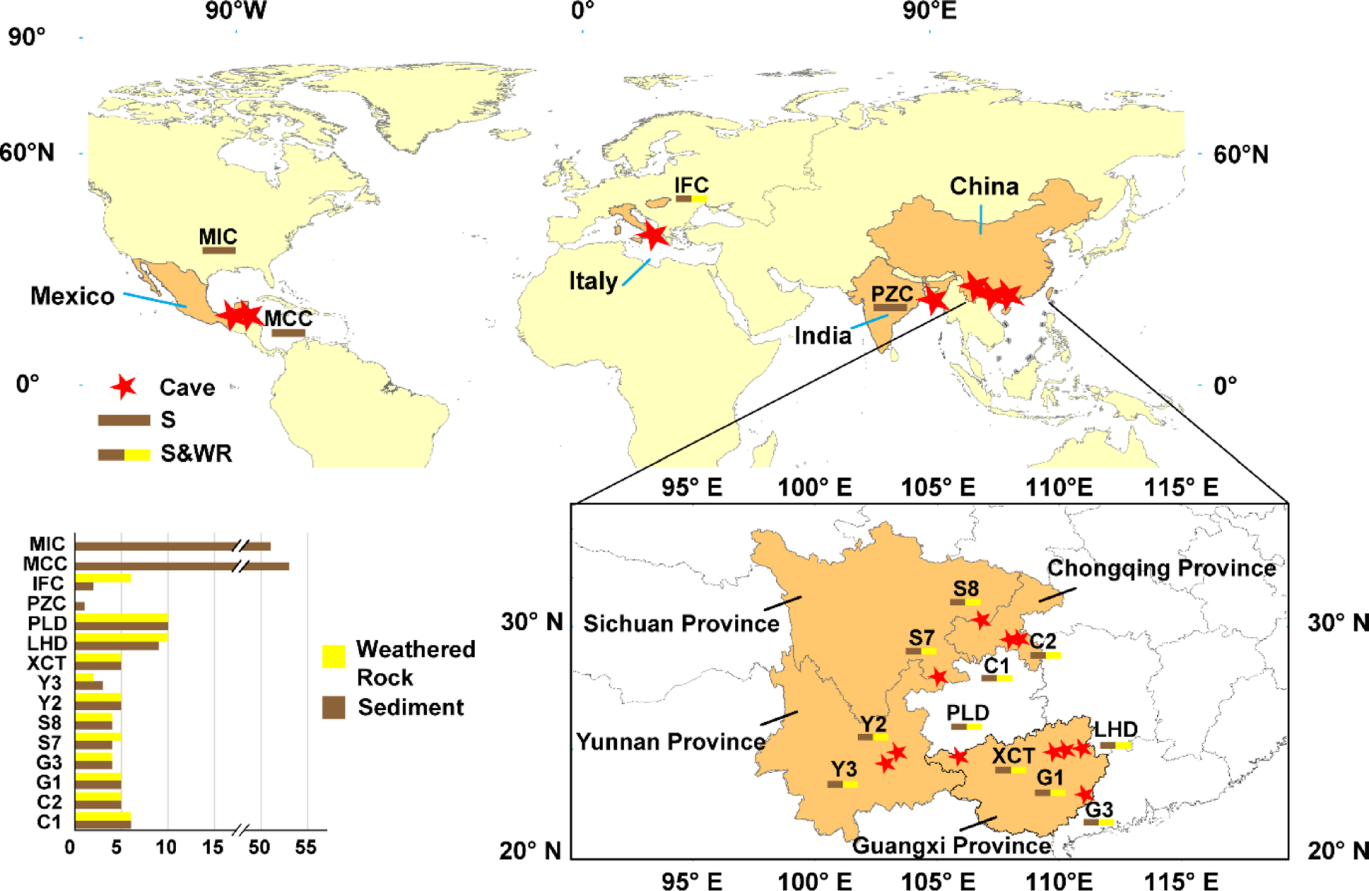
Map of the geographical distribution of the 15 karst caves around the world. Bar graphs in the lower left corner indicate the number of sediment and weathered rock samples within each cave

A total of 147 samples of weathered rock (WR) and sediment (S) were collected from three karst caves in Guilin City (24° 30′–25° 30′ N, 109° 30′–111° 00′ E; WGS84), Guangxi Province, China. They were Panlong Cave (PLD; 22° 56′ 46″ N, 112° 02′ 39″ E; limestone; 30 weathered rock samples and 30 sediment samples), Luohandu Cave (LHD; 25° 0′ 55.8″ N, 110° 54′ 14.2″ E; dolomite, gray dolomite; 27 weathered rock samples and 30 sediment samples), and Xincuntun Cave (XCT; 24° 58′ 38.5″ N, 109° 44′ 15.7″ E; limestone; 15 weathered rock samples and 15 sediment samples) (Fig. 1). At each site, samples were collected using a five-point diagonal sampling strategy. Following homogenization, the samples were stored in sterile 50 mL Corning tubes, sealed, and transported under refrigerated conditions to the Geomicrobiology Laboratory at China University of Geosciences (Wuhan) within 24 h of collection.

A total of 36 weathered rock and 37 sediment samples were provided by collaborating institutions, the Institute of Microbiology, Chinese Academy of Sciences, and included caves from Chongqing (C1, C2), Sichuan (S7, S8), Yunnan (Y2, Y3), and Guangxi (G1, G3) (Fig. 1).

Publicly available 16S rRNA gene sequencing datasets from karst cave environments worldwide were included for comparative analysis. A comprehensive literature search was conducted using databases, such as Web of Science, Elsevier, and ScienceDirect for the period 2015 to 2023 (Fig. 1). Keywords used included “karst cave”, “16S rRNA” and related terms. Studies were selected based on the following criteria: (1) the cave must be a natural cave environment with minimal anthropogenic disturbance; (2) the microbial habitats studied must include weathered rock and/or sediment; (3) the study must focus on bacterial communities; (4) the amplified hypervariable region must include V4 (V4 or V3–V4); and (5) raw sequencing data must be available in the NCBI Sequence Read Archive (https://www.ncbi.nlm.nih.gov/). An additional 6 weathered rock and 107 sediment samples were retrieved from published studies on karst caves in India (PZC) [42], Italy (IFC) [43], and Mexico (MIC, MCC) [44, 45]. These datasets were obtained from 12 caves located across Eurasia and the Americas (20°–40° N, 88°–110° E), encompassing regions characterized by savanna [42, 44, 45], Mediterranean [43], and subtropical monsoon climates [46].

Based on these criteria, raw sequencing data for 333 samples of weathered rock and sediment were gathered for subsequent analysis.

### DNA extraction, PCR amplification, and sequencing

Total genomic DNA samples from PLD, XCT, and LHD was extracted from the samples using the PowerSoil® DNA Isolation Kit (Mo Bio Laboratories, Carlsbad, CA, USA) according to the manufacturer’s instructions. The quality and concentration of the extracted DNA were assessed by 1.0% (w/v) agarose gel electrophoresis and a NanoDrop ND-1000 spectrophotometer (NanoDrop Technologies, Wilmington, DE, USA).

The bacterial 16S rRNA gene V3–V4 hypervariable region was amplified using the primers 338F (5′- ACTCCTACGGGAGGCAGCAG-3′) and 806R (5′-GGACTACHVGGGTWTCTAAT-3′) on an Applied Biosystems (ABI) Veriti™ 96-Well Fast Thermal Cycler [47]. The PCR products were purified using the EZNA® Gel Extraction Kit (Omega Bio-Tek, Norcross, GA, USA). Sequencing libraries were generated and subsequently sequenced on an Illumina MiSeq platform (Illumina, San Diego, CA, USA).

### Physicochemical analysis

The physicochemical properties of weathered rock and sediment samples were analyzed according to the corresponding source of microbial sequencing data for each cave. For samples collected by our research group, weathered rock and sediment were dried, ground, and homogenized with deionized water. The pH was measured using a water quality analyzer (HACH, Loveland, CO, USA), total organic carbon (TOC) content was determined using an elemental analyzer (Vario MACRO cube, Elementar, Germany), and concentrations of major cations, such as K, Ca, Zn, and Mg, were analyzed using inductively coupled plasma optical emission spectrometry (ICP-OES; iCAP 7600 + , Thermo Fisher Scientific). For samples obtained from southwestern China through collaborative institutions, similar pretreatment procedures were applied. pH measurements for these samples were conducted by the respective collaborating laboratories using a benchtop pH meter (FE20, Mettler Toledo, Zurich, Switzerland), while TOC and total nitrogen (TN) contents were analyzed using a TOC/TN analyzer (vario TOC, Elementar, Hanau, Germany). Metallic elements including Ca, K, and Mg were quantified using an elemental analyzer (vario EL III, Elementar, Hanau, Germany). Physicochemical data (including pH, temperature, TOC, and major elements) for the public datasets were directly retrieved from the supplementary materials or results of the original publications [42–45]. Detailed measurement protocols can be found in the respective studies.

### Raw data processing

Raw microbial community sequencing data from karst cave weathered rock and sediment samples were uniformly processed and analyzed using the computing server of our research group. The analysis followed a standardized multi-step workflow. In the first step, raw SRA files were converted into paired-end FASTQ files using the fastq-dump tool, and sequencing data from each cave were individually imported into QIIME 2 (2019.7). In the second step, primer sequences specific to the bacterial V4 hypervariable region-515F (GTGCCAGCMGCCGCGGTAA) and 805R (GACTACHVGGGTATCTAATCC)-were trimmed from each dataset, and the trimmed data were exported for each cave. In the third step, the trimmed sequences from all caves were consolidated into a unified list and re-imported into QIIME 2 using the qiime tools import function. This approach of trimming sequences separately and merging them afterward allowed for greater flexibility in adding or removing datasets. In the fourth step, quality filtering and denoising were performed. The merged dataset was first inspected using the interactive visualization tool QIIME 2 View (https://view.qiime2.org/) to assess sequence quality. Denoising was carried out using the qiime dada2 denoise-paired plugin to remove low-quality reads and chimeric sequences, generating feature tables and representative sequences, along with denoising summaries [48, 49].

The denoised dataset was subsequently subjected to rarefaction, phylogenetic tree construction, and taxonomic annotation to produce the final dataset for downstream analysis. For rarefaction, a sequencing depth that maximized sample retention across sites was selected, and the qiime feature-table rarefy function was used to obtain an amplicon sequence variant (ASV) table. Phylogenetic trees were constructed by exporting representative sequences in FASTA format, performing multiple sequence alignment using FastTree, and subsequently removing hypervariable regions to build both rooted and unrooted phylogenetic trees. For taxonomic annotation, the SILVA reference database (Release 132; http://www.arb-silva.de) was customized within QIIME 2 to match the primer pair 515F/805R. The trained classifier was then applied to the representative sequences to assign bacterial taxonomy and associated classification confidence scores.

After quality filtering and denoising, a total of 14,459,145 high-quality reads were obtained from all samples, resulting in 92,068 ASVs. To minimize biases caused by uneven sequencing depth, all samples were rarefied to 10,000 reads per sample for downstream alpha- and beta-diversity analyses. The rarefaction depth was determined based on alpha-rarefaction curves, which showed that most samples reached a plateau at this sequencing depth, indicating sufficient coverage of microbial diversity.

### Community analysis

#### Community diversity

Microbial community diversity in cave weathered rock and sediment samples was analyzed using the “vegan” package in R (4.3.1). Alpha diversity metrics-including Shannon–Wiener index (H), ASV richness, Chao1 index, ACE index, Pielou’s evenness index (J), and Good’s coverage-were calculated to assess community diversity and sampling completeness. The relative abundances of dominant phyla and genera were statistically analyzed using R. To identify taxa with significant differences between weathered rock and sediment at the genus level, STAMP analysis was performed with a significance threshold of *p* < 0.001. Indicator species associated with varying light exposure levels in rock and sediment samples were identified using LEfSe analysis with an LDA score threshold of 3.5.

#### Distance-decay relationship analysis

Distance-decay relationships of microbial communities from Southwest China, including Panlong Cave, Luohandu Cave, Xincuntun Cave and caves from Chongqing (C1, C2), Sichuan (S7, S8), Yunnan (Y2, Y3), and Guangxi, were analyzed using the “geosphere” package in RStudio (v2025.09.2 + 418). Geographic distances between sampling sites (excluding elevation) were calculated based on latitude and longitude coordinates, and were then compared against community dissimilarities derived from various distance metrics, including Bray–Curtis, Euclidean, Jaccard, distances. Additionally, Mantel tests were conducted using the “linkET” and “dplyr” packages in R to assess the effects of environmental factors on microbial community composition and diversity across different light exposure levels in both rock and sediment habitats.

#### Community assembly

The iCAMP (Infer Community Assembly Mechanisms by Phylogenetic-bin-based null model analysis) approach was applied to investigate the microbial community assembly processes. All computations were conducted on the Galaxy platform (http://ieg3.rccc.ou.edu:8080/), using an OTU abundance table, an unrooted phylogenetic tree, a taxonomy annotation table, and a sample metadata file as inputs. Based on phylogenetic relationships, microbial communities were partitioned into multiple bins, with each bin containing 24 taxa. A significance threshold of *p* < 0.05 was set for detecting differences among taxa. The beta Net Relatedness Index for each bin (βNRIₖ) was calculated and subsequently corrected using the Raup-Crick (RC_K_) metric. Bins with |βNRIₖ|> 1.96 were considered to be governed by deterministic processes, with |βNRIₖ|< −1.96 indicating homogeneous selection (HoS), and |βNRIₖ|> 1.96 indicating heterogeneous selection (HeS). Bins with |βNRIₖ|< 1.96 were attributed to stochastic processes, further classified as homogenizing dispersal (HD) when RC_K_ < −0.95, dispersal limitation (DL) when RCK > 0.95, and drift (DR) when |RCK|< 0.95. Based on the relative abundance of bins in each microbial community, the dominant ecological assembly processes in weathered rock and sediment habitats were inferred. Circular plots displaying bin relative abundances, dominant taxa, and prevailing ecological processes were generated using the “Circular layout” function on the Chiplot platform (https://www.chiplot.online/). Dominant taxa and processes associated with key bins were also statistically summarized. Linear regressions between environmental factor means and community assembly metrics were performed using the “ggplot2” package in R.

#### Network analysis

Molecular network was constructed based on the R package “psych” to calculate the correlations among microorganisms (the top 1% of species with the highest species abundance), with a correlation threshold of R > 0.6 and *p* < 0.01. Molecular ecological networks were visualized and analyzed for topological features using Gephi software (version 0.9.2). All data visualizations involved in the above analyses were completed using the “ggplot2” and “ggpubr” packages in R.

## Results

### Microbial diversity and composition in weathered rock and sediment habitats

**Fig. 2:**
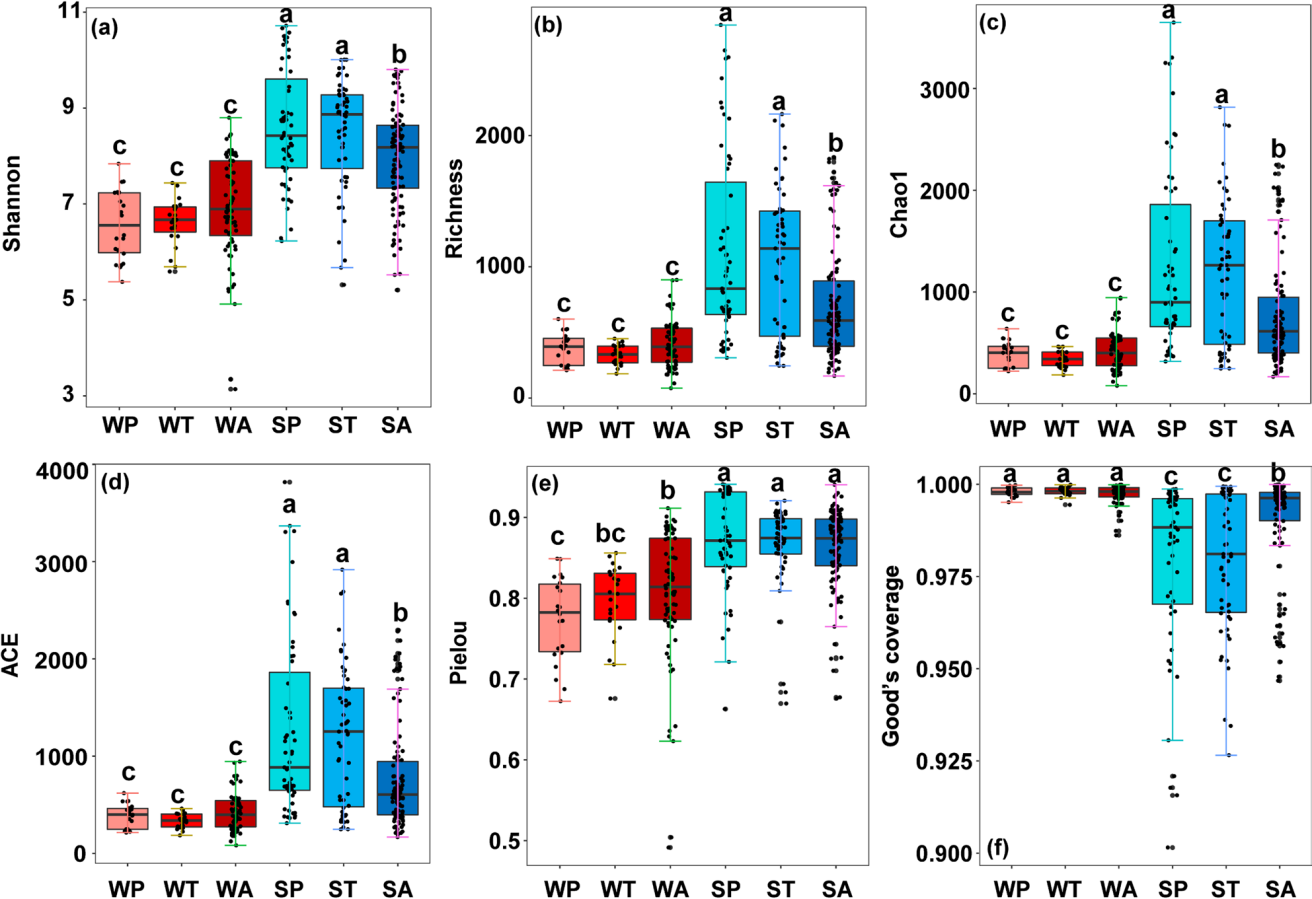
Alpha diversity indexes of the microbial communities in different habitats. **a** Shannon index, **b** Richness index, **c** Chao1 index, **d** ACE index, **e** Pielou index, and **f** Good’s coverage index

Investigated samples were divided into six groups depending on the weathered rock (WR) and sediment (S) habitats and three cave zones. The area closest to the entrance is called the photic zone (P). The middle area inside the cave is called the twilight zone (T). The distant area in the cave is the aphotic zone (A). The microbial diversity varied between the sediment and the weathered rock habitats. Compared with sediment microbial communities, weathered rock communities exhibited lower richness, evenness, and Shannon index values, indicating reduced microbial diversity in weathered rock habitats (Fig. 2a–e). The Good’s coverage indices from all six groups are higher than 0.975, meaning the samples were well represented (Fig. 2f). The distance from the cave entrance had a remarkable effect on the diversity of the sediment microbial community. Sediment aphotic zone had the lowest Shannon index, richness, Chao 1, and ACE index compared with sediment photic and sediment twilight zone (Fig. 2a–d). There was no significant difference in microbial community diversity of weathered rock with different distance from the cave entrance. Therefore, the influence of the distance from the cave entrance was more pronounced in sediment microbial diversity relative to the weathered rock microbial community.

**Fig. 3:**
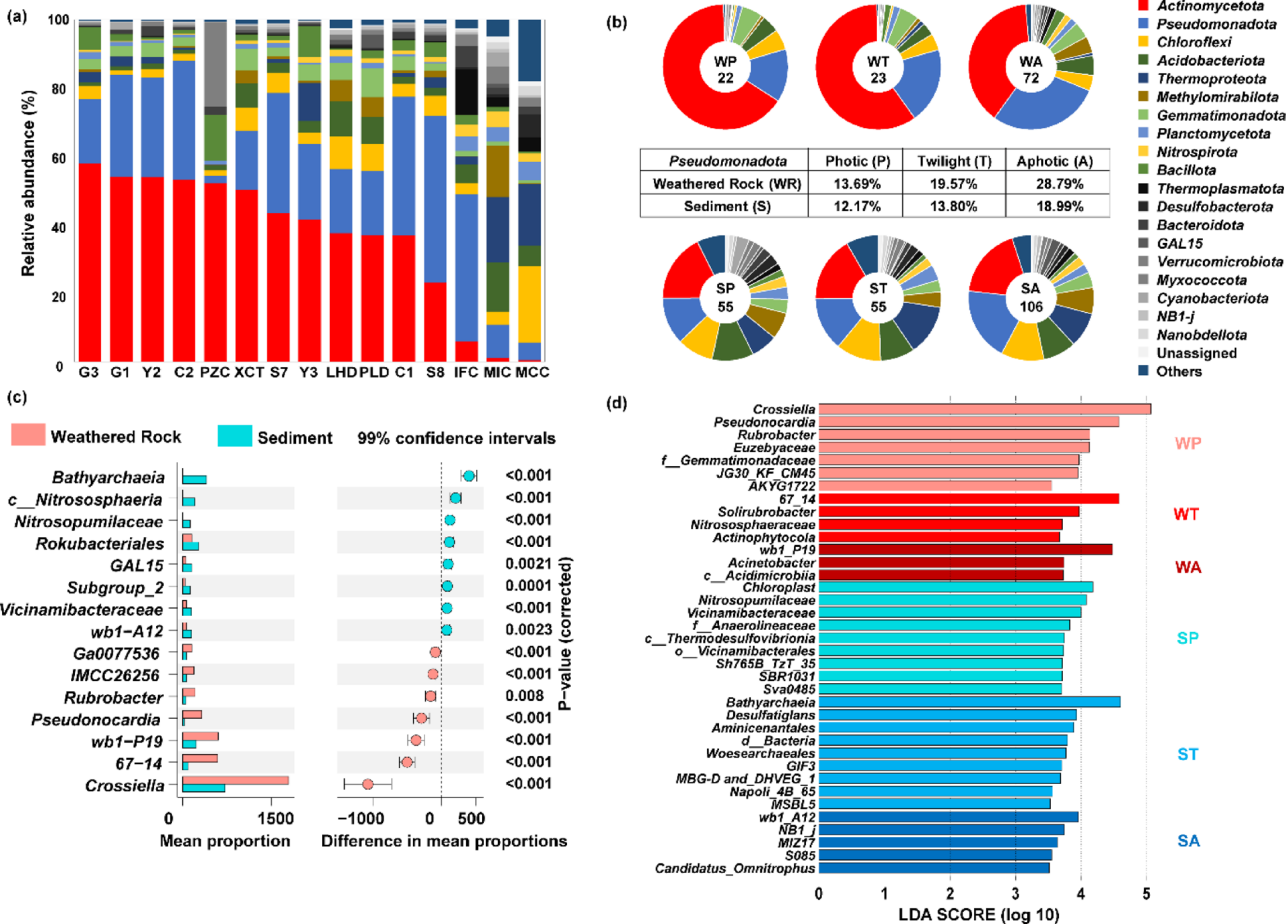
Microbial community composition and indicator taxa in weathered rock and sediment. **a** Relative abundance of the top 20 bacterial phyla across different cave sites. Samples are arranged from left to right according to the decreasing relative abundance of *Actinomycetota*, highlighting variation in dominant phyla among caves. **b** Relative abundance of *Pseudomonadota* from the entrance to the distant part of the cave. **c** Genus-level differential abundance analysis between weathered rock and sediment, performed using STAMP. Only taxa with statistically significant differences are shown. **d** Indicator taxa identified for different microbial community groups based on indicator species analysis. Bar lengths represent indicator values

The relative abundance of the dominant species in the caves was calculated to analyze the composition of microbial communities in different caves. The results elucidated that different caves have similar dominant phyla, while the microbial community composition of underwater caves (MIC and MCC) was significantly different from that of other caves. As shown in Fig. 2a, the top 2 phyla, *Actinomycetota* and *Pseudomonadota,* occupied above 49% of 13 caves (15 caves in total). However, the top 2 phyla were not abundant in underwater caves (MIC and MCC). Notably, the relative abundance of *Thermoproteota* was particularly high (17.94% to 19.26%) in underwater caves, and generally less than 11.11% in other caves (Fig. 3a).

The microbial community composition revealed distinct differences in abundance patterns (Fig. 3b). *Actinomycetota* dominated both habitats, with significantly higher abundance in weathered rock compared to sediment (Fig. 2b). In weathered rock, *Actinomycetota* and *Pseudomonadota* together accounted for over 65% of the total abundance, while other phyla remained less abundant. In contrast, sediment exhibited more homogeneity, with *Actinomycetota* and *Pseudomonadota* comprising less than 40% of the total abundance, and other phyla like *Chloroflexi*, *Acidobacteriota*, and *Thermoproteota* contributing 6.86–13.11% (Fig. 3b). Microbial composition varied noticeably with light change, particularly in weathered rock, where *Pseudomonadota* abundance increased significantly from photic to aphotic zones (Fig. 3b), indicating a negative correlation with light condition. Additionally, as light decreased, *Pseudomonadota* often replaced *Actinomycetota* as the dominant species in both habitats, with more pronounced changes in weathered rock samples and sediment samples (Fig. 3b).

STAMP analysis was displayed to compare the differences of dominant genera and classes in habitats. The results indicated that there were more dominant genera and classes belonging to *Actinomycetota* (such as *Crossiella*, *67_14*, *Pseudonocardia, and Rubrobacter*) and *Pseudomonadota* (such as *Nitrosococcaceae,* and *wb1-P19*) in weathered rock. Archaea (such as *Nitrososphaeria*, *Nitrosopumilaceae,* and *Bathyarchaeia*) and other bacteria (such as *wb1-A12*, *Rokubacteriales*, *Subgroup_2,* and *Vicinamibacteraceae*) were dominant in sediment (Fig. 3c).

LEfSe analysis was used to investigate the indicator species in weathered rock and sediment under photic, disphotic, and aphotic light conditions (Fig. 3d). Indicator species in weathered rock photic zone (such as *Clostridium* spp. and *Pseudonocardia* spp.) and weathered rock twilight zone (such as *Erythrobacter* spp. and *67_14*) were dominated by *Actinomycetota*, and indicator species in weathered rock aphotic zone (such as *Acinetobacter* spp. and *wb1_P19*) were dominated by *Pseudomonadota* (Fig. 3d). The indicator species in sediment photic zone (such as *Vicinamibacteraceae*, *Thermodesulfovibrionia*, and *Anaerolineaceae*) and sediment twilight zone (such as *Bathyarchaeia* and MBG-D/DHVEG-1) were mainly *Chloroflexi* and *Thermoproteota*, and the indicator species in sediment aphotic zone were mainly genera of the *Methylomirabilota* (Fig. 3d).

### Distance decay patterns and the influence of environment factors in weathered rock and sediment and different light zones

**Fig. 4:**
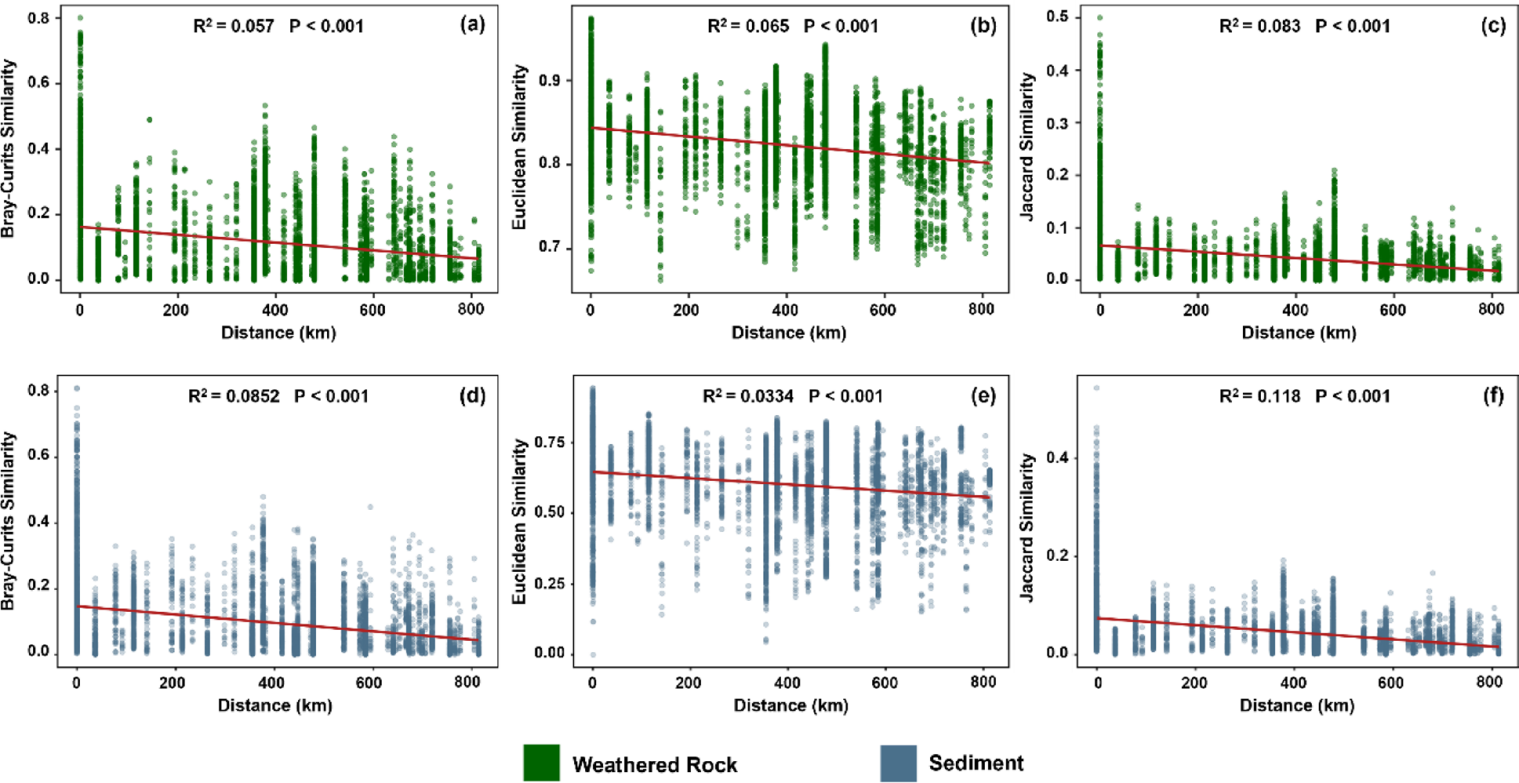
Distance-decay relationships of microbial communities in weathered rock and sediment. Panels **a**–**c** show distance-decay relationships for weathered rock, and panels **d**–**f** for sediment. Community dissimilarity was calculated using Bray–Curtis distance in panels (**a**) and (**d**), Euclidean distance in panels (**b**) and (**e**), and Jaccard distance in panels (**c**) and (**f**)

Across the karst caves from the southwestern region of China, significant distance-decay relationships were observed in both weathered rock and sediment microbial communities (Fig. 4). For weathered rock habitats (Fig. 4a–c), microbial community similarity decreased significantly with increasing geographic distance under all three distance metrics. Bray–Curtis similarity exhibited a clear negative relationship with distance (R^2^ = 0.057, *P* < 0.001), indicating a gradual turnover in community composition. Similar patterns were observed using Euclidean (R^2^ = 0.065, *P* < 0.001) and Jaccard distances (R^2^ = 0.083, *P* < 0.001). For sediment habitats (Fig. 4d–f), distance-decay relationships were also significant (*P* < 0.001 for all metrics), but the explanatory power varied among metrics. Bray–Curtis similarity showed a comparable but slightly stronger distance effect (R^2^ = 0.0852) than that observed in weathered rock, whereas Euclidean distance exhibited a weaker relationship (R^2^ = 0.0334). In contrast, Jaccard distance displayed the strongest distance-decay signal among sediment communities (R^2^ = 0.118), indicating pronounced spatial turnover in taxonomic composition. Notably, the Euclidean similarity was stronger in weathered rock communities than in sediment communities. In contrast, the Jaccard-based distance-decay relationship was stronger in sediment communities than in weathered rock communities.

Mantel test was used to investigate the effects of environmental factors on microbial community composition and diversity in weathered rock and sediment habitats. Generally, environmental factors had more influence on weathered rock microbial communities compared with sediment microbial communities. pH was the most significant environmental factor affecting weathered rock photic zone and weathered rock aphotic zone microbes (Table 1). K and Ca prominently affected the composition and diversity of weathered rock aphotic zone, and C/N and Na were the dominant factors in the sediment aphotic zone. Weathered rock twilight zone microbial community composition and diversity were remarkably influenced by C/N, temperature, Ca, and Na, but sediment twilight zone microbial composition was only significantly affected by TOC (R = 0.875, *p* < 0.01). Microorganisms in weathered rock photic zone and sediment photic zone exhibited a significant difference affected by environmental factors. TOC, C/N, and temperature mainly impacted the microbial community diversity of weathered rock photic zone. However, sediment photic zone microorganisms had no apparent effects from any environmental factors.

**Table 1:** Mantel tests of the effects of environmental factors on the community diversity in each group

Group		TOC	C/N	pH	Temp	K	Ca	Na
	A	0.914	− 0.002	0.930*	0.914	0.912	0.883	0.805
WP(10)	P	0.867*	0.368**	0.871*	0.876*	0.865	0.848	0.820*
	G	0.740*	0.623**	0.735*	0.751*	0.740	0.709	0.692
	A	0.107	0.601*	0.077	0.141	0.311	0.318*	0.496*
WT(8)	P	− 0.047	− 0.046	− 0.194	0.017	− 0.234	− 0.027	− 0.084
	G	− 0.064	0.178	− 0.272	0.502*	− 0.261	0.079	0.045
	A	0.088	0.150	0.281*	− 0.032	0.281*	0.276**	0.034
WA(36)	P	0.002	0.135	0.208*	− 0.072	0.177*	0.207**	0.062
	G	0.023	− 0.049	0.354**	− 0.057	0.265*	0.183**	− 0.002
	A	0.947	0.947	− 0.571	0.947	0.891	0.337	0.836
SP(8)	P	0.949	0.949	0.038	0.949	0.985	0.836	0.336
	G	0.636	0.636	0.565	0.636	0.741	1.000	− 0.217
	A	0.333	0.232	0.428	0.053	− 0.114	− 0.151	0.111
ST(3)	P	0.068	− 0.188	− 0.229	0.345	− 0.197	0.143	− 0.278
	G	0.875**	0.157	0.300	0.350	− 0.190	− 0.210	− 0.156
	A	0.042	0.419**	0.167*	− 0.043	0.181*	− 0.014	0.315**
SA(36)	P	0.161	0.263*	0.184*	0.017	0.1449*	− 0.091	0.473**
	G	− 0.063	− 0.162	− 0.160	0.063	0.075	0.188**	0.039

Group names are followed by the sample size in parentheses. Environmental variables include total organic carbon (TOC, %), sulfur content (S, mg kg^−1^), and temperature (℃). A represents alpha diversity indices, whereas P and G denote the relative abundance of the top ten dominant phyla and genera, respectively. Mantel test results are presented as correlation coefficients (R) and corresponding *p* values. Bold values indicate statistically significant correlations with R > 0.2 and *p* < 0.05. Asterisks indicate significance levels (*p* < 0.05*, *p* < 0.01**)

### Microbial community assembly

**Fig. 5:**
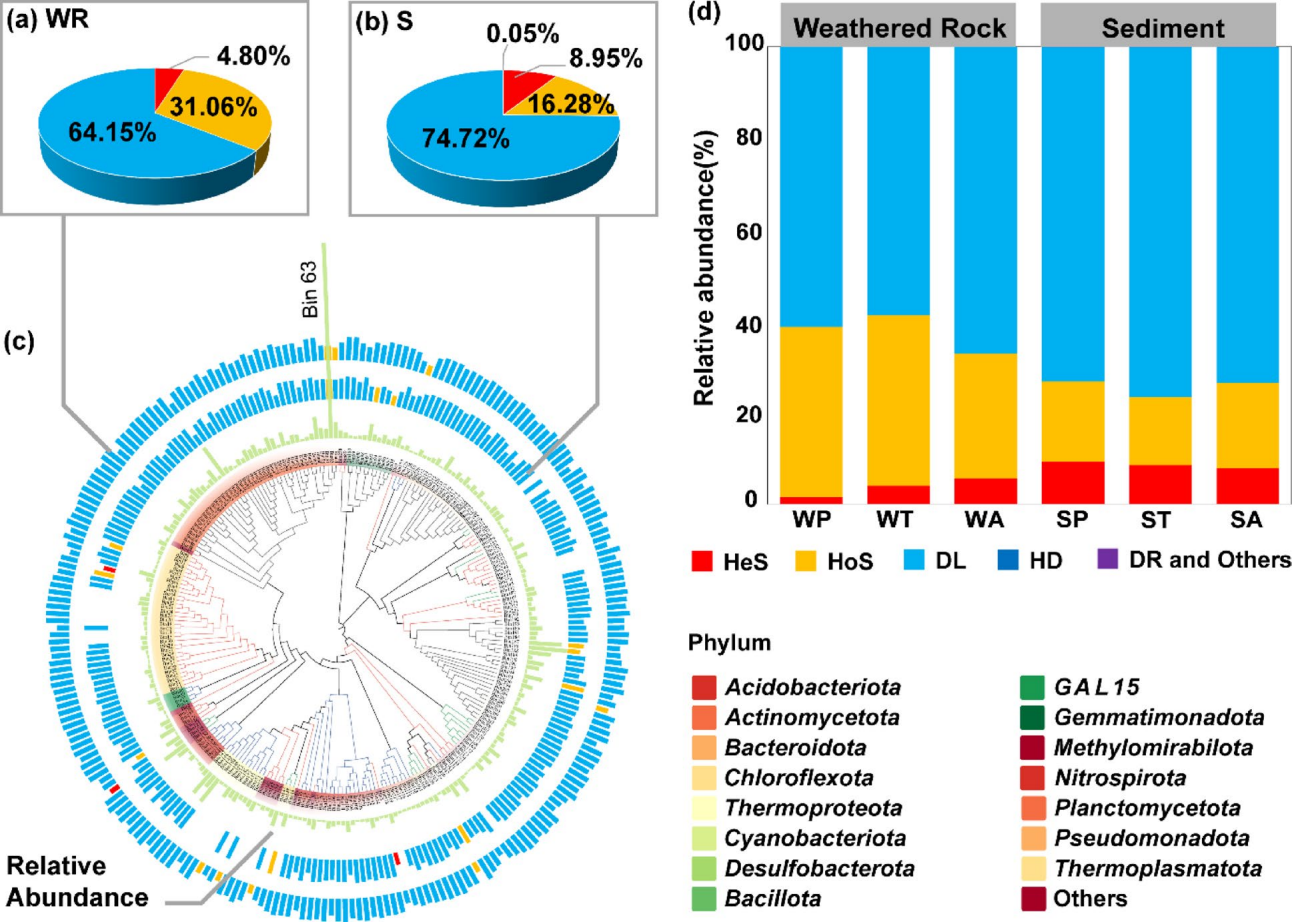
Community assembly mechanisms results of the investigated cave weathered rock and sediment microbial communities and community assembly mechanisms results of different Bins. **a** Weathered rock and **b** sediment habitat microbial community assembly mechanisms results. **c** The circle diagram from outside to inside showed the dominant ecological process and the relative abundance of each Bin in the sediment. The dominant ecological process and the relative abundance of each Bin in weathered rock habitat and relative abundance of each Bin across the community were analyzed. **d** The results of microbial community assembly mechanisms in weathered rock (WR) and sediment (S) habitats and photic (P), twilight (T), and aphotic (A) zones

The principle of null models employed by iCAMP allows us to distinguish between stochastic and deterministic ecological processes in the assembly of bacterial communities in weathered rock and sediment habitats. Specifically, the fractions of homogeneous selection (HoS) and heterogeneous selection (HeS) are considered as deterministic ecological processes, while dispersal limitation (DL), homogenizing dispersal (HD), and drift (DR) are regarded as stochastic ecological processes. Interestingly, commonalities were observed across different habitats in terms of ecological processes. Across the analyzed dataset of caves, stochastic processes were the dominant factor in microbial assembly, irrespective of the specific habitat (weathered rock or sediment) or light conditions (photic, twilight, or aphotic). DL emerged as the primary stochastic process (Fig. 5). Specifically, stochastic processes had greater relative importance in sediment, while deterministic processes had higher significance in weathered rock (Fig. 5d).

Investigated cave microbes were categorized into 233 bins in weathered rock and 251 bins in sediment based on their distant phylogenetic relationships. Among these bins, Bin 63 was the most abundant (Fig. 5c). Analysis of bins with relative abundance greater than 1% revealed dominant bins belonging to *Actinomycetota* (Bin 60, Bin 63, and Bin 76), *Pseudomonadota* (Bin 186, Bin 187, and Bin 188), *Thermoproteota* (Bin 148 and Bin 150), and *Methylomirabilota* (Bin 122) (Table 2). The higher deterministic processes observed in weathered rock microorganisms were primarily due to the higher deterministic processes of the abundant bins compared to sediment (Table 2). Overall, the majority of bins were influenced by DL, accounting for 210 bins (94.17%) in weathered rock and 240 bins (95.62%) in sediment.

**Table 2:** Top 1% relative abundance Bin of weathered rock and sediment

Bin	BinRA (%)	Kindom	Phylum	Genus	Weathered rock	Sediment
Bin 63	10.31	Bacteria	*Actinomycetota*	*Crossiella*	HoS	HoS
Bin 186	2.02	Bacteria	*Pseudomonadota*	*wb1-P19*	HoS	DL
Bin 76	1.84	Bacteria	*Actinomycetota*	*67–14*	DL	DL
Bin 187	1.71	Bacteria	*Pseudomonadota*	*wb1-P19*	HoS	DL
Bin 150	1.52	Archaea	*Thermoproteota*	*c_Nitrososphaeria*	DL	DL
Bin 148	1.40	Archaea	*Thermoproteota*	*Nitrosopumilaceae*	DL	DL
Bin 122	1.34	Bacteria	*Methylomirabilota*	*wb1-A12*	DL	DL
Bin 188	1.19	Bacteria	*Pseudomonadota*	*Ga0077536*	DL	DL
Bin 60	1.18	Bacteria	*Actinomycetota*	*Pseudonocardia*	DL	DL

**Fig. 6:**
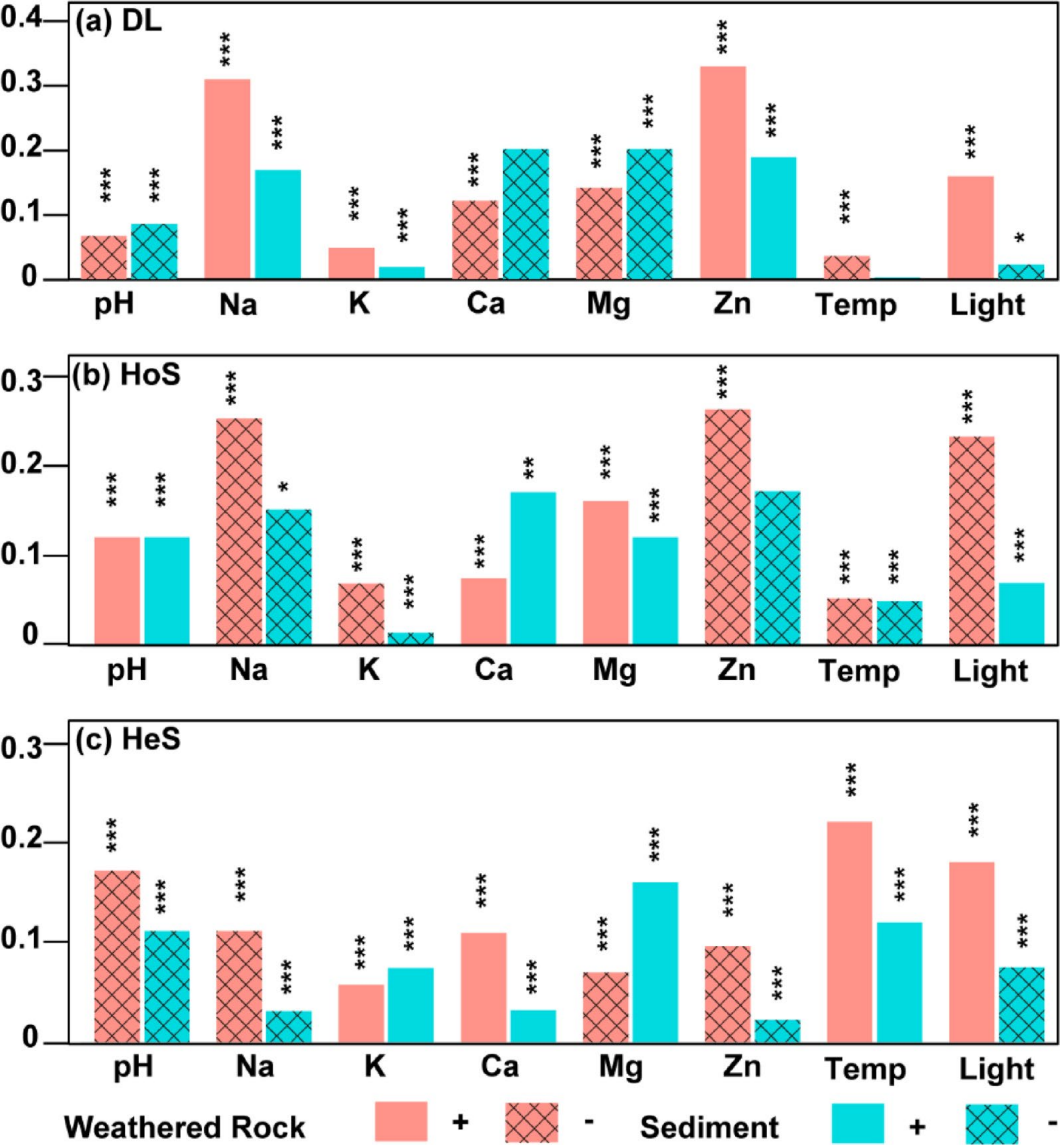
Relationships between microbial community assembly processes and environmental factors in weathered rock and sediment habitats. **a** Associations between environmental factors and dispersal limitation. **b** Associations between environmental factors and homogeneous selection. **c** Associations between environmental factors and heterogeneous selection. Panels (a) represent stochastic assembly processes, whereas panels (b) and (c) represent deterministic assembly processes. The y-axis indicates standardized regression coefficients, reflecting the strength and direction of the relationships. Bars extending above zero (“ + ”) indicate positive correlations, whereas bars extending below zero (“–”) indicate negative correlations. Asterisks denote statistical significance (* *p* < 0.05; ** *p* < 0.01; *** *p* < 0.001)

The impacts of environmental factors on each ecological process were assessed using linear fitting. Overall, environmental factors exhibited significant correlations with both deterministic and stochastic processes. The construction of microbial communities in weathered rock and sediment showed remarkable associations with nearly all environmental factors (*p* < 0.05). The increase in pH and Mg levels was found to decrease the stochastic process. The increase in Na and Zn levels enhanced the stochastic process and weakened the deterministic process (Fig. 6). It is worth noting that the effects of light on the results of weathered rock and sediment community assembly mechanisms are different. In weathered rock, the stochastic processes of microorganisms are enhanced, and the deterministic processes (especially HoS) are weakened when light decreases. In sediment, the stochastic and deterministic processes of microorganisms with reduced light changed little and were more stable than those of weathered rock.

### Co-occurrence networks

**Fig. 7:**
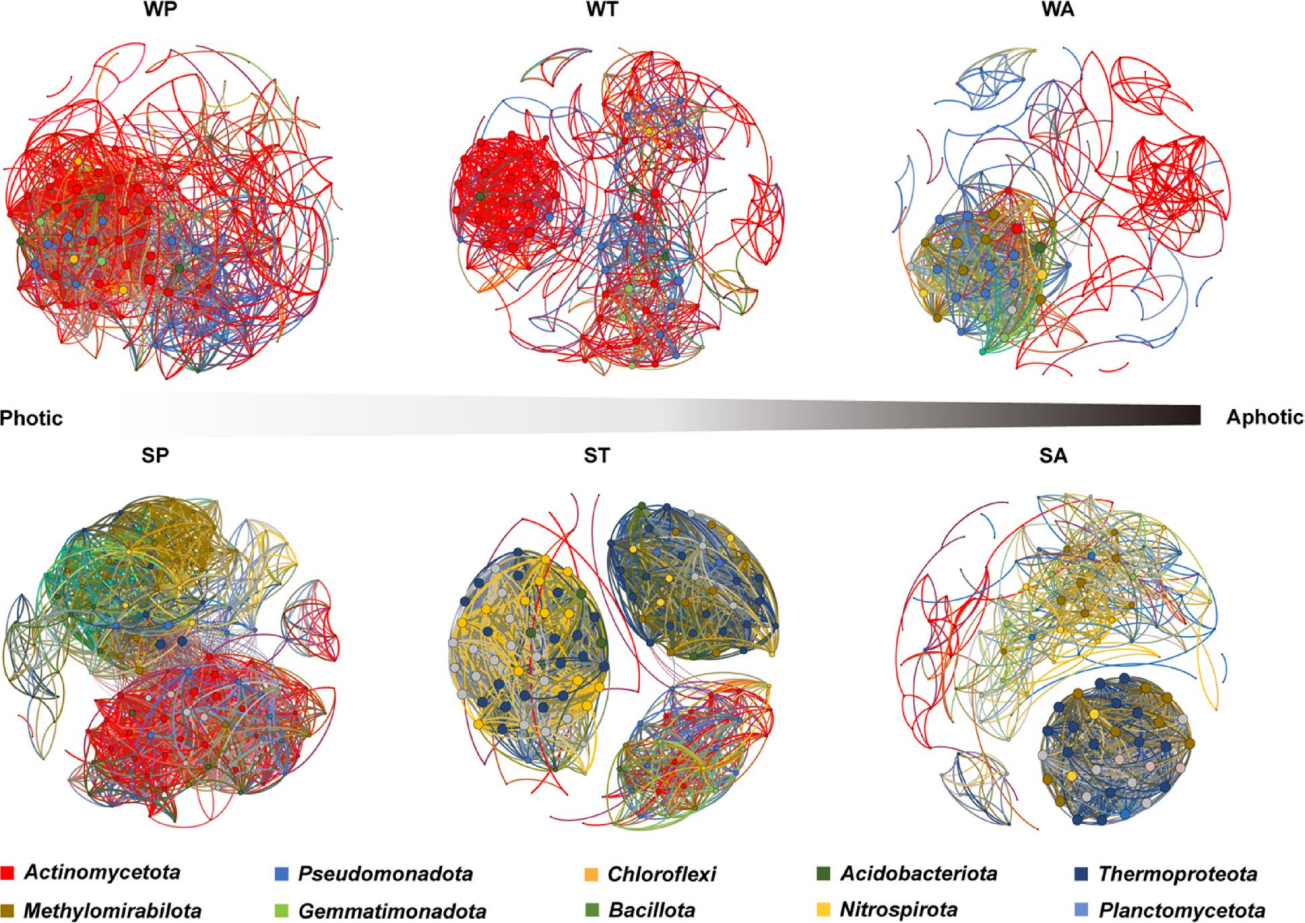
Molecular ecological networks of weathered rock and sediment microbial communities. Nodes in the figure are colored at the phylum level

The analysis was conducted using the top 1% relative abundance of ASVs in the microbial community (Fig. 7). The co-occurrence networks of microbial communities revealed that the sediment microbial network exhibited greater complexity compared to the weathered rock network, except for the sediment aphotic zone network, which had fewer interactions (Table 3). Overall, the sediment microbial network demonstrated higher connectivity among microorganisms. The average degree and average weighted degree of the sediment network were significantly higher than those of the weathered rock network (Table 3). The sediment network displayed stronger connectivity and modularity, as indicated by the higher average clustering coefficient and modularity index compared to the weathered rock microorganisms under the same light condition (Fig. 7). This suggested that sediment network tended to form multiple larger modules to sustain its survival. In contrast, the modularity in the weathered rock network was weaker, with microbial nodes exhibiting limited modularity except for a few larger modules (Fig. 7).

**Table 3:** The topology of weathered rock and sediment network

Group	Node	Line	AVD	Weighted degree	Modularity	ACC	APL
WP	135	1370	20.296	14.376	0.764	0.616	2.635
WT	140	1373	11.457	14.772	0.667	0.622	3.664
WA	117	1140	9.744	14.532	0.474	0.736	3.533
SP	142	2066	29.085	31.876	0.717	0.748	2.372
ST	136	2361	34.721	58.845	0.57	0.917	1.853
SA	128	1005	15.703	26.48	0.492	0.736	3.818

The aphotic zone network exhibited distinct characteristics compared to networks of other distance from the cave entrance. The aphotic zone displayed lower connectivity among microorganisms, and the average weighted degree was lower than that of the photic and twilight zone network (Table 3). The modularity of the aphotic zone network was lower than that of the photic and twilight zone networks (Table 3). These observations suggest a correlation between light condition and the modularity of the network.

## Discussion

### Habitat-dependent diversity patterns and taxonomic succession along spatial gradients

The sediment microbial community demonstrated a higher diversity index and more homogeneous species composition compared to weathered rock, with similar observations having been reported in other caves [7, 14, 18, 50]. The higher alpha diversity in sediment samples may be attributed to the relatively nutrient-rich conditions within oligotrophic caves. Microbial community richness was higher in sediment photic zone and sediment twilight zone than in sediment aphotic zone. A study found that the increase in phototrophic microorganisms led to increased diversity in Carlsbad Cave, Mexico [17]. Therefore, the higher microbial community diversity in sediment photic and sediment twilight may be attributed to the presence of oxygenic phototrophs.

*Actinomycetota* and *Pseudomonadota* were the most abundant phyla in both weathered rock and sediment habitats of various caves, which is consistent with findings from other studies on karst cave microbial communities [15, 51–53]. Other dominant phyla, such as *Chloroflexi* and *Acidobacteriota*, typically have relative abundances exceeding 1%. *Thermoproteota* is more abundant in underground river ecosystems compared to other karst caves, as also observed in other research [8].

The composition of microbial communities did not change substantially as the distance from the cave entrance increased in sediment samples. However, there was a significant increase in the abundance of the phylum *Pseudomonadota* in weathered rock samples, as the distance from the cave entrance increased, gradually replacing some of the phylum *Actinomycetota*, due to the dominance of dark-adapted dominant genera in the phylum *Pseudomonadota* [54, 55]. Similarly, the dominant genera of the *Actinomycetota* phylum, *Rubrobacter*, *Actinobacterium*, *Brevitalea*, and *Gaiella*, decreased from the entrance to the distant zone of the sampling site in weathered rock of Heshang Cave [54]. The variation in microbial indicator species in the weathered rock of caves also indicates the influence of the distance from the cave entrance on the composition of weathered rock microbial communities, with *Actinomycetota* dominating the indicator species in the photic and twilight zones, and *Pseudomonadota* dominating the indicator species in the aphotic zones.

The dominant genera differ significantly between weathered rock and sediment. In weathered rock samples, *Crossiella* is a more significant genus that can interact with other species to support the survival of microbial communities in oligotrophic environments [56, 57], as observed in the microbial studies of Heshang Cave. In sediment samples, the significantly dominant phylum and family are *Thermoproteota*, *Methylomirabilota*, and *Vicinamibacteraceae*. The first two phylum have shown potential methane metabolism [18], while *Vicinamibacteraceae* has been associated with nitrogen fixation capacity [58].

### Distinct spatial turnover in weathered rock and sediment habitats

The distance-decay relationship finding aligned with the general biogeographical rule that dispersal limitation becomes increasingly prominent as geographic distance increases [59], but it also highlighted distinct assembly mechanisms driving community turnover in different cave niches. The higher R^2^ for Jaccard similarity in sediment communities compared with weathered rock communities suggests that spatial separation primarily drives taxonomic turnover in cave sediments. This high heterogeneity in sediments can be attributed to their heterogeneous origins. Cave sediments are primarily composed of surface soil and organic debris transported into the subterranean system via water flow and gravity [60]. Since the surface vegetation and soil properties vary significantly across the geographically distinct karst landscapes of Southwest China [61], the source of the sediments likely imposes a strong legacy on the sediment microbiomes, leading to high compositional dissimilarity between distant caves. The weathered rock communities exhibited more consistent distance-decay patterns across Bray–Curtis, Euclidean, and Jaccard metrics, suggesting that both community composition and relative abundances respond coherently to spatial separation. Specifically, weathered rock communities displayed a relatively lower rate of species turnover but a stable abundance-based structure, as the R^2^ of Euclidean similarity is higher than that of the sediments. This suggests that the weathered rock interface acts as a stronger environmental filter. The carbonate bedrock provides a distinct and relatively consistent oligotrophic substrate across different caves [2]. This ubiquitous geochemical selection may constrain the colonization of diverse transient species, selecting for a core set of specialized lithophilic taxa that are widely shared across the region, thereby buffering the rate of species turnover compared to the highly variable sediment accumulation. The consistently negative slopes across all metrics indicate that geographic distance plays a significant role in structuring microbial communities in karst cave systems, even within a regional spatial scale spanning southwestern China. However, the overall relatively low R^2^ values suggest that spatial distance alone explains only a limited proportion of community variation [62]. This implied that additional factors, particularly local environmental heterogeneity and habitat-specific constraints, may also contribute substantially to community differentiation, which was further assessed using Mantel tests linking microbial dissimilarity with environmental variables.

The effect of environmental factors varied with light conditions. Weathered rock photic zone was significantly influenced by environmental factors. In photic zone (WP and SP), the fluctuation of light will directly affect the growth of photosynthetic microorganisms [63], and the growth of these microorganisms is often limited by changes in these environmental conditions. The microbial communities in the sediment photic zone showed no significant response to environmental factors because sediments act as a buffered environment, reducing exposure to external disturbances. Therefore, microbial communities in sediment are generally relatively more stable [64]. Though in photic zone, the structure of the sediment itself affects the distribution of water and nutrients, making it easier for the microbial community in the sediment to remain stable. Microbes in the aphotic zone being most significantly influenced by environmental factors. The aphotic zone of caves is in total darkness, and microorganisms cannot use sunlight for photosynthesis. Therefore, microbes rely on chemosynthesis for energy [65]. As a result, these microbes in aphotic zones are more sensitive to changes in environmental factors.

### Stochastic processes dominated the community assembly process

Stochastic processes, with dispersal limitation being the prominent factor, play a major role in both cave weathered rock and sediment microbial communities. This finding is consistent with previous studies that have demonstrated the significance of dispersal limitation in microbial communities at regional and global scales [66, 67]. Previous research has demonstrated that increasing spatial distance leads to an intensified effect of dispersion limitation on microbial communities [67, 68]. Abiotic factors primarily constrain microbial patterns at larger spatial and temporal scales, while biotic factors exert a more significant influence on microbial patterns at smaller scales [69]. Dispersal limitation becomes progressively stronger in microbial communities at global scales, making it a more influential factor in shaping microbial communities on a global level [70]. Microbial communities in weathered rock are more strongly affected by light condition, resulting in stronger deterministic processes in weathered rock. This aligns with the observation that the dominant dark-tolerant genus *wb1-P19* exhibits deterministic patterns in weathered rock and stochastic patterns in sediment. The linear relationship between environmental factors and community composition results also indicates a stronger influence of environmental factors on community assembly in weathered rock habitats.

In the investigated weathered rock and sediment microbial communities, the contribution of drift is nearly negligible, which is lower than what has been observed in other studies. This implies that drift plays a minor role in caves with larger spatial extents, and the significance of drift varies depending on the community size, with smaller communities experiencing a greater influence of drift on microbial diversity [71]. Previous research has indicated that the effect of drift is more pronounced in small-scale and fragile habitats [72]. For example, the contribution of small-scale microbial drift process is about 50% in Heshang Cave weathered rock habitats, and the contribution limit of regional scale drift process is reduced, but it is still larger than that of sediment microbial community in Heshang Cave sediment habitat [54]. The relative influence of drift process of microbial communities at regional scale is higher than that at small scale, but it is significantly reduced at large scale, indicating that the effect of scale is different in weathered rocks and sediments.

### Greater network complexity and connectivity in sediment and photic zone communities

Microbial co-occurrence network analysis indicated that network complexity varies with substrate type and the distance from the cave entrance. The higher connectivity and number of lines of the sediment microbial network suggest that microbial communities in sediments tend to form stable subgroups to enhance ecological adaptability [73]. Highly modular structures are often associated with greater ecological robustness, allowing microorganisms to better cope with environmental fluctuations [74, 75]. Additionally, the higher average degree and weighted degree in the sediment network further support stronger microbial interactions, which could facilitate the sharing of key functional genes and metabolic cooperation, thereby improving system stability and resource utilization efficiency [76]. Metabolic exchanges were detected in nutrients-limited environments from previous studies [77, 78], and it was confirmed that metabolic cooperation could drive the co-occurrence pattern and shape the compositions of communities [79]. Therefore, differences in co-occurrence networks between weathered rock and sediment may be because of different interdependencies in the microorganisms [80].

Notably, the distance from the cave entrance also significantly influence the structure of microbial co-occurrence networks. With increasing distance into the cave, both connectivity and modularity decline. In particular, in the aphotic zone, reduced microbial interactions may reflect lower metabolic activity and a stronger habitat filtering effect due to limited energy input [81]. This finding supports the role of light as a critical ecological factor shaping microbial community structure [2]. Moreover, the reduction in microbial cooperation in the distant part of cave zones suggests potential constraints on ecosystem stability and function, as previously observed in marine and soil microbial ecosystems [82].

## Conclusions

Microbial composition and diversity vary among different habitats within karst caves. The sediment microbial community demonstrates a higher diversity index and more homogeneous species composition compared to weathered rock. The observed distance-decay patterns indicate that geographic distance contributes to microbial community differentiation in caves, but its influence is weaker than that of local environmental and habitat-specific controls. The distance from the cave entrance has a notable impact on the composition of microbial communities. Microbial communities in aphotic areas exhibited the weakest distance decay patterns. Photic and disphotic microbial communities showed stronger modularity compared to aphotic zones. The dominant processes shaping microbial communities in caves are primarily stochastic processes. Dispersion limitation prominently influences the investigated cave microbial community, and drift has minimal impact on the karst cave microbial community.

## Data Availability

All raw sequences of new field samples were deposited and available in the NCBI Short Read Archive (SRA) under the project numbers PRJNA1293160 (Panlong Cave), PRJNA1294418 (Luohandu Cave), and PRJNA1294416 (Xincuntun Cave).

## References

[1] Covington MD, Martin JB, Toran LE, et al. Carbonates in the critical zone. Earths Future. 2023;11(1):e2022EF002765.

[2] Wang Y, Cheng X, Wang H, et al. The characterization of microbiome and interactions on weathered rocks in a subsurface karst cave, Central China. Front Microbiol. 2022;13:909494. 35847118 10.3389/fmicb.2022.909494 PMC9277220

[3] Downey AR, Riddell JL, Padilla IY, et al. Storage and distribution of organic carbon in cave sediments: examples from two caves in the northern karst region of Puerto Rico. Environ Earth Sci. 2023;82(1):36. 38840929 10.1007/s12665-022-10720-2 PMC11150972

[4] Mulec J, Pašić L, Oarga-Mulec A. Metabolic traits of sediment bacteria in karst caves in the light of environmental changes. Front Microbiol. 2025;16:1724116. 41459229 10.3389/fmicb.2025.1724116 PMC12742472

[5] Barton H, Taylor N, Kreate M, et al. The impact of host rock geochemistry on bacterial community structure in oligotrophic cave environments. Int J Speleol. 2007;36(2):93–104.

[6] Yun Y, Wang H, Man B, et al. The relationship between pH and bacterial communities in a single karst ecosystem and its implication for soil acidification. Front Microbiol. 2016;7:1955. 28018299 10.3389/fmicb.2016.01955 PMC5159436

[7] Zhu HZ, Zhang ZF, Zhou N, et al. Diversity, distribution and co-occurrence patterns of bacterial communities in a karst cave system. Front Microbiol. 2019;10:1726. 31447801 10.3389/fmicb.2019.01726 PMC6691740

[8] Pašić L, Kovče B, Sket B, et al. Diversity of microbial communities colonizing the walls of a karstic cave in Slovenia. FEMS Microbiol Ecol. 2010;71(1):50–60. 19817862 10.1111/j.1574-6941.2009.00789.x

[9] Cuezva S, Fernandez-Cortes A, Porca E, et al. The biogeochemical role of ctinobacteria in Altamira Cave, Spain. FEMS Microbiol Ecol. 2012;81(1):281–90. 22500975 10.1111/j.1574-6941.2012.01391.x

[10] Wu Y, Tan L, Liu W, et al. Profiling bacterial diversity in a limestone cave of the western Loess Plateau of China. Front Microbiol. 2015;6:244. 25870592 10.3389/fmicb.2015.00244 PMC4378288

[11] Ortiz M, Neilson JW, Nelson WM, et al. Profiling bacterial diversity and taxonomic composition on speleothem surfaces in Kartchner Caverns, AZ. Microb Ecol. 2013;65(2):371–83. 23224253 10.1007/s00248-012-0143-6

[12] Wang Y, Jiao M, Zhao Z, et al. Insight into the role of niche concept in deciphering the ecological drivers of MPs-associated bacterial communities in mangrove forest. Water Res. 2024;249:120995. 38071907 10.1016/j.watres.2023.120995

[13] Gogoleva N, Chervyatsova O, Balkin A, et al. Microbial tapestry of the Shulgan-Tash cave (Southern Ural, Russia): influences of environmental factors on the taxonomic composition of the cave biofilms. Environ Microb. 2023;18(1):82. 10.1186/s40793-023-00538-1 PMC10662634 37990336

[14] Xu W, Liao L, Liao D, et al. Distribution of carbon-sequestering microbes in different habitats and the interaction with habitat factors in a natural karst cave. Sustainability. 2024;16(17):7357.

[15] Bogdan DF, Baricz AI, Chiciudean I, et al. Diversity, distribution and organic substrates preferences of microbial communities of a low anthropic activity cave in North-Western Romania. Front Microbiol. 2023. 10.3389/fmicb.2023.962452. 36825091 10.3389/fmicb.2023.962452 PMC9941645

[16] Park S, Cho YJ, Jung D, et al. Microbial diversity in moonmilk of Baeg-nyong Cave, Korean CZO. Front Microbiol. 2020;11:613. 32390967 10.3389/fmicb.2020.00613 PMC7190796

[17] Havlena Z, Kieft TL, Veni G, et al. Lighting effects on the development and diversity of photosynthetic biofilm communities in Carlsbad Cavern, New Mexico. Appl Environ Microbiol. 2021;87(6):e02695–20. 33452019 10.1128/AEM.02695-20 PMC8105000

[18] Jia A, Jianeng G, Yancheng L, et al. The diversity of microbes and prediction of their functions in karst caves under the influence of human tourism activities—a case study of Zhijin Cave in Southwest China. Environ Sci Pollut Res. 2022;29(17):25858–68. 10.1007/s11356-021-17783-x 34854002

[19] Liu W, Xu M, Kattel GR, et al. Deterministic and Stochastic processes regulate co-occurrence network structure and shape macroinvertebrate diversity in Karst Environment. Ecol Evol. 2026;16(1):e72802. 41509570 10.1002/ece3.72802 PMC12774801

[20] Bontemps Z, Moënne-Loccoz Y, Hugoni M. Stochastic and deterministic assembly processes of microbial communities in relation to natural attenuation of black stains in Lascaux Cave. mSystems. 2024;9(2):e01233–23. 38289092 10.1128/msystems.01233-23 PMC10878041

[21] Tang X, Xie G, Shao K, et al. Contrast diversity patterns and processes of microbial community assembly in a river-lake continuum across a catchment scale in Northwestern China. Environ Microb. 2020;15(1):10. 10.1186/s40793-020-00356-9 PMC8066441 33902721

[22] Stegen JC, Lin X, Konopka AE, et al. Stochastic and deterministic assembly processes in subsurface microbial communities. ISME J. 2012;6(9):1653–64. 22456445 10.1038/ismej.2012.22 PMC3498916

[23] Zhang ZF, Mao J, Cai L. Dispersal limitation controlling the assembly of the fungal community in Karst caves. J Fungi. 2023;9(10):1013. 10.3390/jof9101013 PMC10608104 37888269

[24] Zhou J, Liu W, Deng Y, et al. Stochastic assembly leads to alternative communities with distinct functions in a bioreactor microbial community. MBio. 2013;4(2):10. 10.1128/mbio.00584-12. 10.1128/mBio.00584-12 PMC3585448 23462114

[25] Yuan C, Wang H, Dai X, et al. Effect of Karst microhabitats on the structure and function of the rhizosphere soil microbial community of *Rhododendron pudingense*. Sustainability. 2023;15(9):7104.

[26] Xu Q, Vandenkoornhuyse P, Li L, et al. Microbial generalists and specialists differently contribute to the community diversity in farmland soils. J Adv Res. 2021;40:17–27. 36100325 10.1016/j.jare.2021.12.003 PMC9481938

[27] Li D, Ni H, Jiao S, et al. Coexistence patterns of soil methanogens are closely tied to methane generation and community assembly in rice paddies. Microbiome. 2021;9:20. 33482926 10.1186/s40168-020-00978-8 PMC7825242

[28] Leibold MA, Govaert L, Loeuille N, et al. Evolution and community assembly across spatial scales. Annu Rev Ecol Evol Syst. 2022;53(1):299–326.

[29] Kopp J, Leveau LM. Bird metacommunities of urban parks in the Pampean region, Argentina. Landsc Urban Plan. 2025;253:105202.

[30] Zhao L, Xiao R, Zhang S, et al. Environmental specificity of karst cave habitats evidenced by diverse symbiotic bacteria in Opiliones. BMC Ecol Evol. 2024;24(1):58. 38720266 10.1186/s12862-024-02248-9 PMC11080181

[31] Thompson PL, Guzman LM, De Meester L, et al. A process‐based metacommunity framework linking local and regional scale community ecology. Ecol Lett. 2020;23(9):1314–29. 32672410 10.1111/ele.13568 PMC7496463

[32] de Paula CCP, Bichuette ME, Seleghim MHR. Nutrient availability in tropical caves influences the dynamics of microbial biomass. MicrobiolOpen. 2020;9(7):e1044. 10.1002/mbo3.1044 PMC7349172 32394640

[33] Bahram M, Hildebrand F, Forslund SK, et al. Structure and function of the global topsoil microbiome. Nature. 2018;560(7717):233–7. 30069051 10.1038/s41586-018-0386-6

[34] Bahram M, Espenberg M, Pärn J, et al. Structure and function of the soil microbiome underlying N_2_O emissions from global wetlands. Nat Commun. 2022;13(1):1430. 35301304 10.1038/s41467-022-29161-3 PMC8931052

[35] Lange-Enyedi NT, Borsodi AK, Németh P, et al. Habitat-related variability in the morphological and taxonomic diversity of microbial communities in two Hungarian epigenic karst caves. FEMS Microbiol Ecol. 2023;99(12):fiad161. 38066687 10.1093/femsec/fiad161

[36] Bukelskis D, Dabkeviciene D, Lukoseviciute L, et al. Screening and transcriptional analysis of polyketide synthases and non-ribosomal peptide synthetases in bacterial strains from Krubera-Voronja Cave. Front Microbiol. 2019;10:2149. 31572349 10.3389/fmicb.2019.02149 PMC6753585

[37] Reitschuler C, Spötl C, Hofmann K, et al. Archaeal distribution in moonmilk deposits from Alpine caves and their ecophysiological potential. Microb Ecol. 2016;71(3):686–99. 26790864 10.1007/s00248-015-0727-z

[38] Biagioli F, Coleine C, Delgado-Baquerizo M, et al. Outdoor climate drives diversity patterns of dominant microbial taxa in caves worldwide. Sci Total Environ. 2024;906:167674. 37813267 10.1016/j.scitotenv.2023.167674

[39] Wiseschart A, Pootanakit K. Chapter 23—Metagenomic-based approach to a comprehensive understanding of cave microbial diversity[M]//DE MANDAL S, BHATT P. Recent Advancements in Microbial Diversity. Academic Press, 2020: 561–586[2024-12-20].

[40] Biagioli F, Coleine C, Piano E, et al. Microbial diversity and proxy species for human impact in Italian karst caves. Sci Rep. 2023;13:689. 36639707 10.1038/s41598-022-26511-5 PMC9839721

[41] Kieft B, Finke N, McLaughlin RJ, et al. Genome-resolved correlation mapping links microbial community structure to metabolic interactions driving methane production from wastewater. Nat Commun. 2023;14:5380. 37666802 10.1038/s41467-023-40907-5 PMC10477309

[42] Iquebal MA, Passari AK, Jagannadham J, et al. Microbiome of Pukzing Cave in India shows high antimicrobial activity against plant and animal pathogens. Genomics. 2021;113(6):4098–108. 34699904 10.1016/j.ygeno.2021.10.004

[43] Cirigliano A, Mura F, Cecchini A, et al. Active microbial ecosystem in iron-age tombs of the Etruscan civilization. Environ Microbiol. 2021;23(7):3957–69. 33200556 10.1111/1462-2920.15327

[44] Santillán J, López-Martínez R, Aguilar-Rangel EJ, et al. Microbial diversity and physicochemical characteristics of tropical karst soils in the northeastern Yucatan peninsula, Mexico. Appl Soil Ecol. 2021;165:103969.

[45] Suárez-Moo P, Remes-Rodríguez CA, Márquez-Velázquez NA, et al. Changes in the sediment microbial community structure of coastal and inland sinkholes of a karst ecosystem from the Yucatan peninsula. Sci Rep. 2022;12(1):1110. 35064185 10.1038/s41598-022-05135-9 PMC8782880

[46] Qian W, Ding T, Hu H, et al. An overview of dry–wet climate variability among monsoon-westerly regions and the monsoon northernmost marginal active zone in China. Adv Atmos Sci. 2009;26(4):630–41.

[47] Claesson MJ, O’Sullivan O, Wang Q, et al. Comparative analysis of pyrosequencing and a phylogenetic microarray for exploring microbial community structures in the human distal intestine. PLoS ONE. 2009;4(8):e6669. 19693277 10.1371/journal.pone.0006669 PMC2725325

[48] Bolyen E, Rideout JR, Dillon MR, et al. Reproducible, interactive, scalable and extensible microbiome data science using QIIME 2. Nat Biotechnol. 2019;37(8):852–7. 31341288 10.1038/s41587-019-0209-9 PMC7015180

[49] Callahan BJ, McMurdie PJ, Rosen MJ, et al. DADA2: high-resolution sample inference from Illumina amplicon data. Nat Methods. 2016;13(7):581–3. 27214047 10.1038/nmeth.3869 PMC4927377

[50] Liu X, Wang H, Wang W, et al. Nitrate determines the bacterial habitat specialization and impacts microbial functions in a subsurface karst cave. Front Microbiol. 2023;14:1115449. 10.3389/fmicb.2023.1115449. 36846803 10.3389/fmicb.2023.1115449 PMC9947541

[51] Vásquez-Castro F, Wicki-Emmenegger D, Fuentes-Schweizer P, et al. Diversity pattern and antibiotic activity of microbial communities inhabiting a karst cave from Costa Rica. Microbiology. 2024;170(11):001513. 39530301 10.1099/mic.0.001513 PMC11555687

[52] Cheng X, Xiang X, Yun Y, et al. Archaea and their interactions with bacteria in a karst ecosystem. Front Microbiol. 2023;14:1068595. 36814573 10.3389/fmicb.2023.1068595 PMC9939782

[53] Ahamada Rachid N, Doğruöz GN. Major impacts of caving activities on cave microbial diversity: case study of Morca Cave, Turkey. Int Microbiol. 2022;26(2):179–90. 36331653 10.1007/s10123-022-00287-0

[54] Ma L, Huang X, Wang H, et al. Microbial interactions drive distinct taxonomic and potential metabolic responses to habitats in karst cave ecosystem. Microbiol Spect. 2021;9(2):e01152-e1221. 10.1128/Spectrum.01152-21 PMC8557908 34494852

[55] Frey B, Walthert L, Perez-Mon C, et al. Deep soil layers of drought-exposed forests harbor poorly known bacterial and fungal communities. Front Microbiol. 2021. 10.3389/fmicb.2021.674160. 34025630 10.3389/fmicb.2021.674160 PMC8137989

[56] Sanchez-Moral S, Portillo MC, Janices I, et al. The role of microorganisms in the formation of calcitic moonmilk deposits and speleothems in Altamira Cave. Geomorphology. 2012;139–140:285–92.

[57] Gogoleva N, Chervyatsova O, Balkin A, et al. Microbial tapestry of the Shulgan-Tash cave (Southern Ural, Russia): influences of environmental factors on the taxonomic composition of the cave biofilms. Environ Microb. 2023;18(1):82. 10.1186/s40793-023-00538-1 PMC10662634 37990336

[58] Niu H, Yuan M, Chen X, et al. Deciphering the differences of bacterial communities between high- and low-productive wheat fields using high-throughput sequencing. Front Microbiol. 2024. 10.3389/fmicb.2024.1391428. 39296300 10.3389/fmicb.2024.1391428 PMC11408337

[59] Wang Y, Lu G, Yu H, et al. Meadow degradation increases spatial turnover rates of the fungal community through both niche selection and dispersal limitation. Sci Total Environ. 2021;798:149362. 34375268 10.1016/j.scitotenv.2021.149362

[60] Ravn NR, Michelsen A, Reboleira ASPS. Decomposition of organic matter in caves. Front Ecol Evol. 2020;8:554651.

[61] Zhang J, Chen H, Fu Z, et al. Effects of vegetation restoration on soil properties along an elevation gradient in the karst region of southwest China. Agric Ecosyst Environ. 2021;320:107572.

[62] Varliero G, Lebre PH, Adams B, et al. Biogeographic survey of soil bacterial communities across Antarctica. Microbiome. 2024;12(1):9. 38212738 10.1186/s40168-023-01719-3 PMC10785390

[63] Imada T, Yamamoto C, Toyoshima M, et al. Effect of light fluctuations on photosynthesis and metabolic flux in *Synechocystis* sp. PCC 6803. Biotechnol Prog. 2023;39(3):e3326. 36700527 10.1002/btpr.3326

[64] Wang L, Hu T, Li Y, et al. Unraveling the interplay between antibiotic resistance genes and microbial communities in water and sediments of the intensive tidal flat aquaculture. Environ Pollut. 2023;339:122734. 37838320 10.1016/j.envpol.2023.122734

[65] Ren M, Jones B, Nie X, et al. Carbonate microbialites and chemotrophic microbes: Insights from caves from South–East China. Sedimentology. 2024;71(5):1558–90.

[66] Li Y, Ma K, Song W, et al. Environmental heterogeneity and dispersal limitation simultaneously determine the spatial scaling of different microbial functional groups. Sci Total Environ. 2023;885:163854. 37142009 10.1016/j.scitotenv.2023.163854

[67] Zhang S, Li K, Hu J, et al. Distinct assembly mechanisms of microbial sub-communities with different rarity along the Nu River. J Soils Sediments. 2022;22(5):1530–45.

[68] Du X, Gu S, Zhang Z, et al. Spatial distribution patterns across multiple microbial taxonomic groups. Environ Res. 2023;223:115470. 36775088 10.1016/j.envres.2023.115470

[69] Xu X, Wang N, Lipson D, et al. Microbial macroecology: in search of mechanisms governing microbial biogeographic patterns. Glob Ecol Biogeogr. 2020;29(11):1870–86.

[70] Ladau J, Eloe-Fadrosh EA. Spatial, temporal, and phylogenetic scales of microbial ecology. Trends Microbiol. 2019;27(8):662–9. 31000488 10.1016/j.tim.2019.03.003

[71] Gilbert B, Levine JM. Ecological drift and the distribution of species diversity. Proc R Soc B Biol Sci. 2017;284(1855):20170507. 10.1098/rspb.2017.0507 PMC5454268 28566486

[72] Vilmi A, Gibert C, Escarguel G, et al. Dispersal–niche continuum index: a new quantitative metric for assessing the relative importance of dispersal versus niche processes in community assembly. Ecography. 2021;44(3):370–9.

[73] Newman MEJ. Modularity and community structure in networks. Proc Natl Acad Sci. 2006;103(23):8577–82. 16723398 10.1073/pnas.0601602103 PMC1482622

[74] Santolini M, Barabási AL. Predicting perturbation patterns from the topology of biological networks. Proc Natl Acad Sci. 2018;115(27):E6375–83. 29925605 10.1073/pnas.1720589115 PMC6142275

[75] Chen W, Wen D. Archaeal and bacterial communities assembly and co-occurrence networks in subtropical mangrove sediments under *Spartina alterniflora* invasion. Environ Microb. 2021;16(1):10. 10.1186/s40793-021-00377-y PMC8091715 33941277

[76] Deng Y, Jiang YH, Yang Y, et al. Molecular ecological network analyses. BMC Bioinf. 2012;13(1):113. 10.1186/1471-2105-13-113 PMC3428680 22646978

[77] Morris BEL, Henneberger R, Huber H, et al. Microbial syntrophy: interaction for the common good. FEMS Microbiol Rev. 2013;37(3):384–406. 23480449 10.1111/1574-6976.12019

[78] Hom EFY, Murray AW. Niche engineering demonstrates a latent capacity for fungal-algal mutualism. Science. 2014;345(6192):94–8. 24994654 10.1126/science.1253320 PMC4409001

[79] Zelezniak A, Andrejev S, Ponomarova O, et al. Metabolic dependencies drive species co-occurrence in diverse microbial communities. Proc Natl Acad Sci. 2015;112(20):6449–54. 25941371 10.1073/pnas.1421834112 PMC4443341

[80] Dong Y, Gao J, Wu Q, et al. Co-occurrence pattern and function prediction of bacterial community in Karst cave. BMC Microbiol. 2020;20(1):137. 32471344 10.1186/s12866-020-01806-7 PMC7257168

[81] Letunic I, Bork P. Interactive Tree Of Life (iTOL) v5: an online tool for phylogenetic tree display and annotation. Nucleic Acids Res. 2021;49(W1):W293–6. 33885785 10.1093/nar/gkab301 PMC8265157

[82] Berry D, Widder S. Deciphering microbial interactions and detecting keystone species with co-occurrence networks. Front Microbiol. 2014;5:219. 24904535 10.3389/fmicb.2014.00219 PMC4033041

